# Probable Acute Hepatic Porphyria Diagnosed Using Urinary Porphyrin Spectrophotometry in a Resource-Limited Setting: A Case Report

**DOI:** 10.7759/cureus.102474

**Published:** 2026-01-28

**Authors:** Mihiran Thanigasalan

**Affiliations:** 1 Internal Medicine, National Hospital of Sri Lanka, Colombo, LKA

**Keywords:** acute porphyria, carbohydrate loading, hoesch test, resource-limited diagnosis, soret peak

## Abstract

Acute hepatic porphyrias are rare metabolic disorders that often present with recurrent neurovisceral symptoms and are frequently misdiagnosed. Definitive diagnosis relies on biochemical and genetic testing, which may be unavailable in resource-limited settings. We report a 36-year-old woman with recurrent episodes of severe colicky abdominal pain associated with vomiting and bowel disturbances, with minimal abdominal findings and repeatedly normal routine investigations. During an acute episode, urinary screening demonstrated a positive Hoesch test for porphobilinogen (PBG). Spectrophotometric analysis of urine total porphyrins revealed a Soret peak at approximately 405 nm, and the total urine porphyrin concentration was elevated at 349.635 nmol/L (reference: <300 nmol/L). Alternative causes of elevated urinary porphyrins were excluded clinically and biochemically. Due to the unavailability of intravenous hemin, the acute episode was managed with analgesia, supportive care, and carbohydrate, leading to symptom resolution. The patient was counselled on the avoidance of precipitating factors and long-term surveillance. This case illustrates the diagnostic value of clinical suspicion supported by basic biochemical testing in identifying probable acute hepatic porphyria in a resource-limited setting.

## Introduction

The porphyrias are a group of inherited metabolic disorders resulting from enzymatic defects in the heme biosynthesis pathway. Acute hepatic porphyrias are characterized by episodic neurovisceral attacks, commonly presenting with severe abdominal pain, autonomic instability, neuropsychiatric manifestations, and minimal objective findings on physical examination or routine investigations [1,2]. Because of their rarity and nonspecific presentation, diagnosis is often delayed, increasing the risk of serious neurological complications.

Acute hepatic porphyrias result from enzymatic defects in the hepatic heme biosynthesis pathway, leading to the accumulation of toxic heme precursors, particularly porphobilinogen (PBG) and aminolevulinic acid (ALA). During acute attacks, overproduction of PBG is a key biochemical abnormality and forms the basis of diagnostic testing. Detection of elevated urinary PBG, therefore, plays a central role in establishing the diagnosis of an acute porphyria episode.

Biochemical confirmation during an acute attack typically demonstrates increased urinary PBG, with or without elevation of ALA and porphyrins [1,3]. In many low- and middle-income countries, access to quantitative biochemical assays and genetic testing is limited, necessitating reliance on clinical features and basic laboratory methods.

## Case presentation

A 36-year-old woman, married for 13 years, presented with a three-day history of acute onset, moderate to severe colicky central and lower abdominal pain associated with nausea, vomiting, and loose stools. She reported intermittent constipation over the preceding seven months.

She had experienced multiple similar episodes in the past requiring hospital admissions, previously treated as constipation and ureteric colic. Less severe attacks had occurred intermittently without medical consultation. There was no history of fever, gastrointestinal bleeding, alcohol use, lead exposure, or relevant family history. She had no known chronic medical illnesses.

On examination, the patient was hemodynamically stable. Abdominal examination revealed a soft, nondistended, nontender abdomen with no organomegaly or palpable masses. There were no features of autonomic dysfunction, such as hypertension, tachycardia, or excessive sweating, observed during the acute episode. Neurological examination was unremarkable. Mental state assessment demonstrated mild depressive symptoms without psychosis. 

Routine laboratory investigations, including complete blood count, inflammatory markers, renal and liver function tests, electrolytes, arterial blood gas analysis, cortisol level, and urinalysis, were within normal limits apart from mild neutrophilic leukocytosis (Table 1). Abdominal ultrasonography and X-ray of the kidneys, ureters, and bladder (KUB) were normal.

**Table 1:** Summary of investigations LDH: lactate dehydrogenase

Investigation	Patient value	Reference range
White blood cell count	12,200/µL	4,000-11,000/µL
Neutrophils	78.6%	40-75%
Lymphocytes	16.9%	20-45%
Hemoglobin	13.3 g/dL	12.0-15.5 g/dL
Mean corpuscular volume (MCV)	88.4 fL	80-100 fL
Platelet count	375,000/µL	150,000-400,000/µL
C-reactive protein (CRP)	<5 mg/L	<5 mg/L
Erythrocyte sedimentation rate (ESR)	22 mm/1st hour	<20 mm/1st hour
Serum creatinine	59 µmol/L	44-97 µmol/L
Blood urea	12 mg/dL	7-20 mg/dL
Random blood glucose	104 mg/dL	70-140 mg/dL
Serum sodium	142 mmol/L	135-145 mmol/L
Serum potassium	4.0 mmol/L	3.5-5.0 mmol/L
Alanine aminotransferase (ALT)	24 U/L	<35 U/L
Aspartate aminotransferase (AST)	28 U/L	<35 U/L
Alkaline phosphatase (ALP)	97 U/L	40-129 U/L
Gamma-glutamyl transferase (GGT)	30 U/L	<38 U/L
Total bilirubin	3.3 µmol/L	3-17 µmol/L
Direct bilirubin	1.7 µmol/L	<5 µmol/L
Serum albumin	4.2 g/dL	3.5-5.0 g/dL
Arterial blood pH	7.44	7.35-7.45
pCO₂	36.9 mmHg	35-45 mmHg
HCO₃⁻	22.2 mmol/L	22-26 mmol/L
Serum LDH	210 U/L	140-280 U/L
Serum amylase	73 U/L	30-110 U/L
9 a.m. serum cortisol	432 nmol/L	123-626 nmol/L
Urinalysis	Normal	No protein, blood, or ketones

Given the recurrent severe abdominal pain with minimal objective findings and neuropsychiatric features, acute porphyria was suspected. Screening for porphyria was performed approximately six days after symptom onset, while the patient was still symptomatic. Urinary total porphyrins were quantified using spectrophotometric analysis of urine samples, and results are reported consistently in nmol/L. Urinary PBG (Hoesch test) was positive. Urine total porphyrins were elevated to 349.635 nmol/L (reference: <300 nmol/L). Spectrophotometry revealed the presence of an absorption peak at approximately 405 nm (Soret peak), consistent with porphyrin compounds [4].

**Figure 1:**
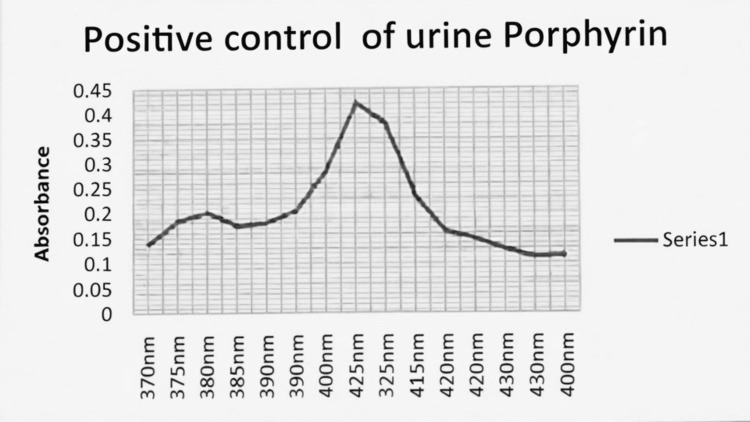
Urinary porphyrin spectrophotometry: positive control Spectrophotometric absorbance spectrum of a positive control urine sample demonstrating a characteristic Soret peak at approximately 405 nm, consistent with the presence of porphyrin compounds. The x-axis represents wavelength (370-440 nm) and the y-axis represents absorbance

**Figure 2:**
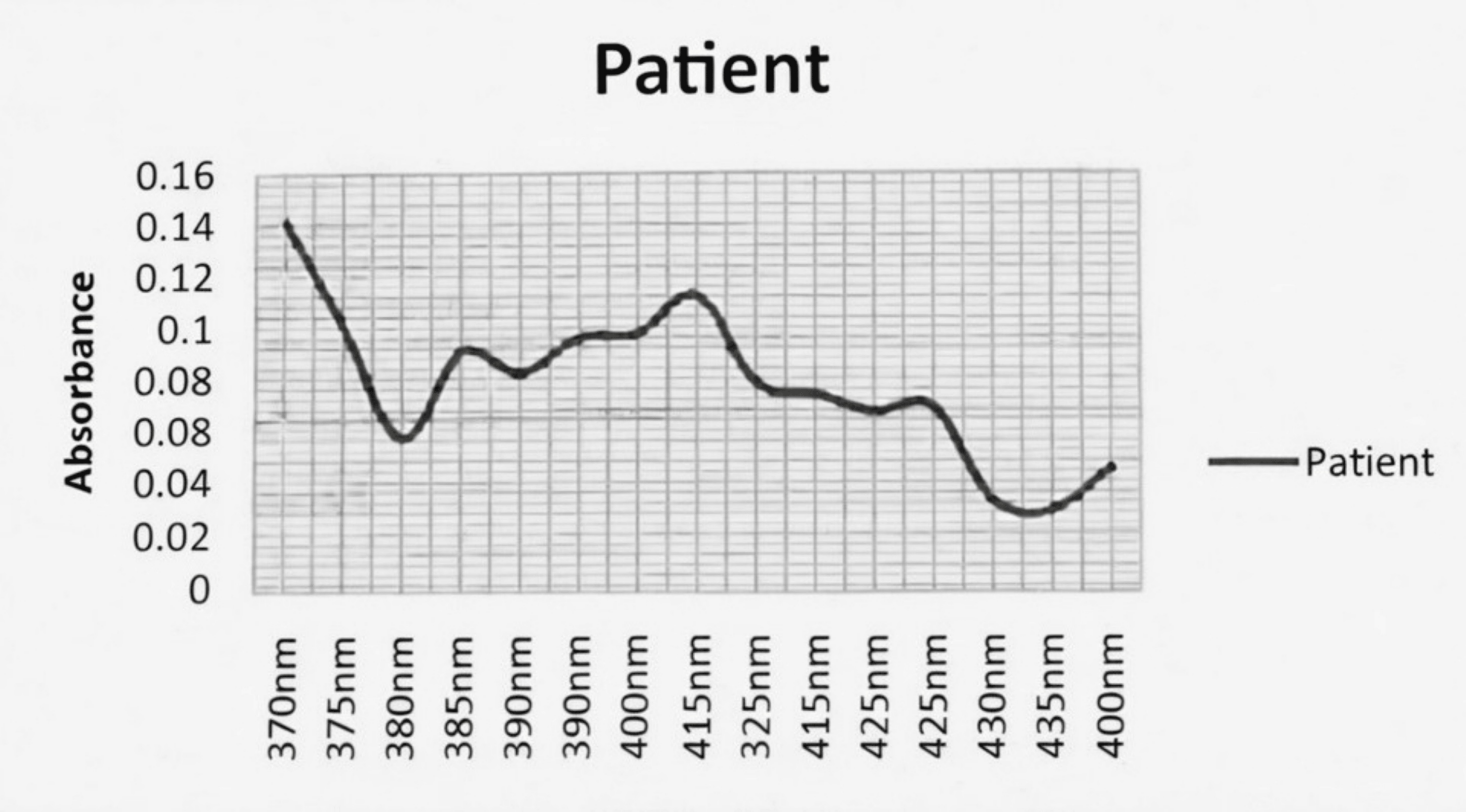
Urinary porphyrin spectrophotometry: patient sample Spectrophotometric absorbance spectrum of the patient’s urine sample showing an absorption peak at approximately 405 nm (Soret peak), consistent with the presence of urinary porphyrins

The patient’s spectrophotometric curve demonstrated a Soret peak similar in wavelength to the positive control (Figure 1), although with lower amplitude and additional minor peaks (Figure 2). These findings are consistent with increased urinary porphyrins and may reflect drug metabolites or sample heterogeneity. Alternative causes of elevated urinary porphyrins, including hepatobiliary disease, hemolysis, alcohol misuse, and lead toxicity, were excluded clinically and biochemically.

Based on the characteristic clinical presentation, positive urinary PBG screening, elevated urine porphyrin concentration, and presence of a Soret peak, a diagnosis of probable acute hepatic porphyria presenting with an acute neurovisceral attack was made [1,3]. Quantitative urinary PBG/ALA measurements and genetic testing were not available.

Intravenous human hemin was not available. The patient was managed with adequate analgesia (paracetamol and subcutaneous morphine), a high-calorie carbohydrate-rich diet, intravenous 10% dextrose (2 L daily for three days), and avoidance of potential porphyrinogenic drugs.

The patient’s abdominal pain and gastrointestinal symptoms resolved with supportive therapy. She was educated regarding the nature of the disease, avoidance of triggering medications, the importance of adequate carbohydrate intake during mild attacks, and the hereditary implications. She was advised long-term follow-up, including periodic abdominal ultrasonography, given the recognized increased risk of hepatocellular carcinoma in acute hepatic porphyrias.

The patient was followed up for six months after discharge. During this period, no recurrence of severe acute attacks was reported, and repeat porphyria-specific biochemical testing was not performed due to limited availability.

## Discussion

This case highlights the classical presentation of an acute porphyria attack, which includes severe recurrent abdominal pain disproportionate to physical findings, normal routine investigations, and associated neuropsychiatric symptoms. Such presentations often result in repeated misdiagnoses and delayed recognition.

During acute attacks, demonstration of excess urinary PBG is the cornerstone of diagnosis [1,3]. Marked elevation of urinary PBG is required to establish the diagnosis of an acute hepatic porphyria attack, whereas isolated elevation of urinary porphyrins is nonspecific and must be interpreted in conjunction with clinical features and PBG testing. In this patient, a positive Hoesch test, elevated total urinary porphyrins, and a characteristic Soret peak provided biochemical support for the diagnosis. While quantitative PBG and ALA measurements and genetic confirmation are required for definitive classification and family screening, current recommendations accept biochemical evidence obtained during an attack as sufficient for diagnosing an acute porphyria episode. Accordingly, the diagnosis in this case is best classified as probable acute hepatic porphyria, given the absence of genetic confirmation.

Management was constrained by a lack of access to hemin therapy. Although carbohydrate loading is inferior to hemin and supported by limited evidence, it remains a pragmatic option in resource-limited settings and was associated with clinical improvement in this patient [1,5].

The absence of quantitative urinary PBG and ALA measurements and the lack of genetic testing limit definitive subtype classification [3]. Nevertheless, this case demonstrates that clinically meaningful diagnosis and management of acute porphyria are achievable using basic biochemical tools when advanced testing is unavailable.

## Conclusions

Acute porphyria should be considered in patients with recurrent severe abdominal pain and minimal objective findings after exclusion of common causes. In resource-limited settings, careful clinical evaluation combined with basic urinary porphyrin testing can support timely diagnosis, allowing appropriate management and prevention of serious complications.

## References

[1] Anderson KE (2019). Acute hepatic porphyrias: current diagnosis & management. Mol Genet Metab.

[2] Balwani M, Desnick RJ (2012). The porphyrias: advances in diagnosis and treatment. Blood.

[3] Sardh J, Harper P (2005). Acute Intermittent Porphyria. https://www.ncbi.nlm.nih.gov/books/NBK1193/.

[4] Puy H, Gouya L, Deybach JC (2010). Porphyrias. Lancet.

[5] Anderson KE, Collins S (2006). Open-label study of hemin for acute porphyria: clinical practice implications. Am J Med.

